# An ethical defense of safer supply

**DOI:** 10.1007/s11017-026-09767-0

**Published:** 2026-07-15

**Authors:** Arthur Krieger

**Affiliations:** Department of Philosophy, Temple University, Philadelphia, PA USA

**Keywords:** Public health, Harm reduction, Evidence-based medicine, Addiction, Risk/benefit analysis, Informed consent

## Abstract

Safer supply is a medical form of harm reduction in which a patient is prescribed a drug to which they are addicted. Despite ample high-quality evidence that it is a safe and effective last-line treatment for severe, treatment-refractory substance use disorders, safer supply is not widely used or advocated. This is likely due in part to ethical concerns, but I contend that safer supply is ethical by both general moral standards and the professional ethical standards of healthcare. Recent defenses of various harm reduction interventions argue that they are permissible regardless of the moral quality of the drug use they facilitate. I critique those defenses and argue that safer supply is permissible by general moral standards because the medical drug use it supports is not immoral. Furthermore, safer supply does not run afoul of biomedical ethics because it is not maleficent, unhealthful, or nonconsensual. As an evidence-supported and ethically permissible practice, safer supply merits wider use and advocacy.

## Introduction

“Safer supply” is the practice of prescribing to a person who uses drugs (PWUD) a drug that they use. In heroin maintenance therapy, for instance, a medical provider prescribes pharmaceutical heroin to someone who uses heroin. Like needle exchange programs, supervised injection sites, and opioid agonist therapies, safer supply is a form of “harm reduction” (HR) for drug use because it aims to mitigate the harms associated with its primary alternative—the illicit procurement and use of street drugs—without eliminating risky drug use altogether [1]. The drugs provided in safer supply are unadulterated, precisely dosed pharmaceutical products, and are provided in the context of the fiduciary patient-provider relationship [2]. These factors reduce the risks of negative health outcomes for patients who otherwise use street drugs, including worsened addiction, addiction to adulterants, torturous withdrawal, overdose, and death [3]. Furthermore, by providing a licit drug supply and medical supervision, medical providers reduce the legal and social risks associated with illicit drug use, including conviction, incarceration, social stigma, and alienation from the healthcare system [4]. Many of the greatest harms of drug addiction are due to the fact that illicit drug supplies are especially unsafe; safer supply responds to this fact. Safer supply can be administered in various ways, including as a take-home regimen [4] or on an outpatient basis [5].

All forms of harm reduction for drug use are controversial because they do not insist that PWUD completely desist [6]. But some forms of HR, like methadone maintenance, have become widely accepted, and even controversial forms, like supervised injection sites, are championed by major harm reduction organizations [7–9]. By contrast, safer supply has received little attention in public discourse, medicine, or HR advocacy, despite growing evidence that it can be safe and effective. This may be because it is viewed as ethically problematic or politically inexpedient. I will argue that safer supply is ethical by both general moral standards and the professional ethical standards of healthcare, i.e. biomedical ethics. As such, I believe it deserves more attention as a last-line harm reduction measure for drug addiction, which for present purposes I take to be equivalent to severe (rather than mild or moderate) substance use disorder (SUD) [10].

My argument will proceed as follows. In the first section, I will give some background on safer supply as an HR strategy, including evidence for its safety and efficacy. In the second section, I will specify the form of safer supply that I will defend, which adheres to the usual standards of medical care, especially best practices in medication management. In the third section, I will counter arguments that safer supply transgresses general moral standards, and in particular, arguments that it involves complicity in immoral drug use or shields PWUD from deserved negative consequences. And in the fourth section, I will counter arguments that safer supply runs afoul of professional biomedical ethics. Specifically, I will address arguments that safer supply harms patients, that it facilitates unhealthy lifestyles, and that people with addictions are not competent to consent to it.

## Background on safer supply

For over a century, physicians have recognized a distinct role for the safer supply of drugs of addiction, which enables patients to avoid withdrawal by medical means and to focus on other aspects of recovery, while avoiding the complications that come with substituting other drugs like methadone [11]. Drucker and colleagues explain that:

in Great Britain… doctors had the authority to gradually detoxify or maintain addicts by prescribing their drugs of choice, including opiates… The United States had a history of morphine maintenance in the 1920 s, as did the United Kingdom, but unlike Britain, American authorities and the American Medical Association were hostile to this approach and shut down these programmes in 1926 [2, pp. 241–242].

After decades of punitive drug policy around the world driven by the US [12], interest in safer supply re-emerged in the late twentieth century. It was a small component in the influential Merseyside Harm Reduction Model implemented in the UK in the 1980 s, which sought to reduce the risk of HIV infection for people who injected heroin [13]. Several studies have been done on safer supply in the UK, Switzerland, Germany, the Netherlands, Canada, and Spain, including randomized controlled trials (RCTs) and large observational studies comparing supervised outpatient heroin administration with that of methadone and morphine [5, 14–23]. A review of all such RCTs conducted internationally as of 2012 found that outpatient heroin maintenance compares favorably to outpatient methadone and morphine therapies with respect to retention, the use of street drugs, physical and mental health outcomes, and criminal activity [3, pp. 44–54]. These results were confirmed by an independent Cochrane review (included in [3, pp. 55–79])and have since been replicated in Canadian studies [22, 23]. Furthermore, a qualitative assessment of ten opioid maintenance pilot projects in Canada (focusing on hydromorphone, hydrocodone, and fentanyl) found that they helped participants to stabilize their lives, address psychiatric comorbidities, get work and housing, break free from hustling and survival sex work, and improve overall wellbeing and safety [4]. High-quality empirical evidence that the safer supply of opioids can be safe and effective is abundant. This evidence should inform both clinical decision-making in evidence-based addiction medicine whenever possible, as well as priorities for further research, including research into the safety and efficacy of safer supply for non-opioid drug classes.

Despite good reason to view the safer supply of opioids as a valuable tool in addiction medicine, it is not widely practiced, and it is not emphasized or explicitly endorsed by leading HR organizations [7–9]. This is notable, given the severity of the international opioid crisis. Equally notable is the relative lack of interest in research on or practice of safer supply for non-opioid drugs, on an inpatient, outpatient, or take-home basis. An exception is the Government of Canada, which endorses safer supply of opioids, benzodiazepines, and amphetamines for take-home use, for the corresponding substance use disorders (SUDs) [24]. But in principle, inpatient, outpatient, and take-home safer supply can be practiced for any drug of addiction that can be medically prescribed, including commonly misused drugs like cocaine, methamphetamine, and ketamine [24]. These forms of safer supply are not yet supported directly by high quality evidence, and further research will likely show that not all forms of safer supply have a favorable risk–benefit analysis. But the probable explanations for the utility of heroin maintenance likely apply to other drug classes as well: Safer supply enables PWUD to avoid severe withdrawals and the harms associated with a more dangerous supply, while facilitating connections to other treatments through improved relations with the healthcare system.

Given the long-running controversy around HR, I hypothesize that safer supply receives less attention than other HR strategies because it is seen as especially ethically problematic or especially politically inexpedient. Below, I will focus on the former: I will argue that evidence-supported safer supply can be ethically permissible by the standards of both general moral considerations and biomedical ethics. Medical providers ought to be free to prescribe opioids and perhaps other drugs when they determine, on the basis of good science, that it is medically indicated, likely to mitigate the harms of drug use, and otherwise comports with their clinical judgment. Where high quality scientific evidence is absent for the safer supply of non-opioid drug classes, further research promises to improve addiction medicine substantially. Where political forces are a major obstacle to advocacy for safer supply practice and research, efforts should be made to mollify them. The ethical arguments presented below may be helpful in such efforts.

## Legal drug dealers? Best practices in safer supply

For those unfamiliar with safer supply as a medical intervention, it is reasonable to worry that it makes healthcare providers into drug dealers and debases the practice of medicine. I should be clear from the outset that my defense will focus on safer supply that adheres to established medical standards, including good medication management. I will not give an exhaustive account of best practices in prescribing, or in medicine generally. But I will highlight some salient features of ethically defensible safer supply. Best practices in safer supply will vary depending on the patient, drug, setting (i.e. inpatient, outpatient, take-home), and other factors, but general features distinguish defensible safer supply from medical negligence or mere drug dealing.

Safer supply ought to be provided only following (1) formal diagnosis with an appropriate addiction-related condition (e.g. severe opioid use disorder) and (2) careful risk–benefit analysis, (3) as a last-line treatment for a patient whose condition is treatment-refractory. These conditions derive from the medical provider’s duty to promote what is in the patient’s best interests, often called “the principle of beneficence” [25, ch. 6]. Like many medical interventions, safer supply has significant risks, including worsened dependence and overdose. When a patient has access to safer alternatives that seem likelier to benefit them, these ought to be tried before safer supply. The ideal safer supply patient is like the typical subject in RCTs of safer supply: someone who has tried all available alternatives and continues to struggle with severe, harmful substance use. Crucially, in the absence of a formal diagnosis establishing the severity of the patient’s condition, it is impossible to make an evidence-based judgment about what interventions are medically indicated.

Once a provider has determined that a patient is a good candidate for safer supply, responsible administration requires active medication management in the context of a strong patient-provider relationship. While the most basic form of safer supply is drug prescription, it is incumbent on providers to monitor patient health status and adherence throughout treatment, and to adjust the prescription dynamically. One major concern in safer supply is dose escalation, including increased supplementation of the prescribed supply with street drugs. A concern in take-home treatment is diversion or “leakage,” particularly the resale of pharmaceuticals at a premium to obtain a greater quantity of street drugs. Dose escalation and diversion both undermine safer supply’s guiding aim of reducing the risks of drug use. Providers must be sensitive to these forms of nonadherence and do their best to minimize them. In some cases, providers can monitor adherence with urinalysis [26]. But the patient is the provider’s primary source of information about their treatment, and patient-provider communication grounded in trust is important for treatment monitoring. This can be challenging with PWUD, who have a reputation for dishonesty and manipulative behavior. But the provider is in a position to cultivate epistemic humility and trust, and to encourage honesty through a non-judgmental and non-punitive approach [27, 28]. Providers may opt to use prescription contracts–sometimes called “opioid contracts” in the context of opioid therapy–to enhance adherence. But contract-based prescribing can weaken trust in the patient-provider relationship, as can the random (as opposed to regular) drug testing they often stipulate [29, 30].

In some contexts, it may be impossible to provide safer supply that adheres to best practices outside of a specially designed program, or without access to special resources. Formal assessment for suitability, dynamic treatment, and adherence monitoring may impose clinical, administrative, or logistical burdens that are prohibitive for medical providers, even providers of addiction medicine. Since I will only defend safer supply that adheres to the medical standards and best practices described above, and since medicine must often be practiced under non-ideal conditions, it might seem that I am defending safer supply as an idealized practice, merely in principle. But many medical interventions must be administered with care at every step, including comparable harm reduction strategies (e.g. opioid agonist therapies) and a wide range of other pharmacotherapies. In my view, safer supply is not distinctive in requiring special measures to ensure that treatment is medically appropriate, and my ethical defense of safer supply is a defense of an actual practice that has been implemented in many contexts and can be readily proliferated where policy allows. Furthermore, my defense of safer supply need not and will not specify every policy, procedure, or mechanism by which high quality treatment can be ensured. As with many other treatments, providers should exercise their expert judgment in determining whether and how to administer safer supply in accordance with the scientific and ethical expectations of modern medicine.

## Moral objections to safer supply

Now that I have identified responsibly administered safer supply as my focus, I can turn to defending it as ethically permissible. I will argue that safer supply is permissible according to two sets of ethical standards: general moral considerations, and biomedical ethics. The general moral considerations to which I refer are ethical objections that any reasonable member of the public might have to safer supply, deriving from values that are widely if not universally shared in modern societies. (I use the term ‘moral’ for its association with universal ethical standards.) The biomedical standards to which I refer are the established ethical standards of healthcare professions, especially medicine. I will begin by addressing two general moral objections to HR that apply to safer supply: (1) that it is complicit in the immoral behavior of people who use drugs, and (2) that it wrongly shields PWUD from negative consequences they deserve to suffer, due to their immoral behavior. Recent defenses of HR practices have argued that they are ethically permissible even if addictive drug use is immoral. I will show that these defenses are inadequate, and that safer supply is permissible because the addictive drug use it enables is not immoral.

## Complicity

One of the main objections to HR services is that they involve complicity in the immoral behavior of recipients. A party is complicit in another party’s wrongdoing when they participate in that conduct such that they are ethically culpable. Since many view drug use as immoral, there is reasonable concern that HR providers are complicit in immoral conduct.

Timothy Kirschenheiter and John Corvino argue that even if drug use is immoral, the operation of supervised injection sites (SISs) and needle exchange programs (NEPs) do not involve complicity, at least not on a consequentialist picture in which wrongdoing is defined in terms of resulting harms [31]. They rely on Gregory Mellema’s analysis of complicity, on which complicity can be due to either ‘enabling,’ ‘facilitating,’ or ‘condoning’ of a harm [32]. To enable a harm is to contribute a key ingredient to its production. If Red sells Pat a gun to murder Zed and Pat murders Zed, then Red is complicit. Crucially, this is true even if Pat could have gotten a gun from someone else or used a kitchen knife they already had; Pat did not need Red to provide a key ingredient, but Red did, and that makes them complicit [31, pp. 355–356]. To facilitate a harm is to make it more likely to occur [31, p. 357]. If Red tells Pat a few locations where Zed might be found, then they increase Pat’s likelihood of killing Zed, making them complicit. Finally, to condone a harm is to explicitly or implicitly endorse the production of a harm [31, p. 358]. If Red encourages Pat to murder Zed, then they condone this harm, and are complicit.

Kirschenheiter and Corvino argue that SISs and NEPs do not enable, facilitate, or condone drug use, and therefore do not entail any complicity in it, regardless of its moral quality. With regard to condoning, our authors claim that these HR services are compatible with a neutral moral assessment of drug use, and do not inherently approve of it [31, pp. 358–360]. With regard to facilitating, they cite studies showing that NEPs and SISs do not increase the prevalence of drug use, which they take to mean that these services do not increase the likelihood of drug use [31, pp. 357–358]. And with regard to enabling, our authors argue that the goods provided by SISs and NEPs–a safe place to inject drugs, and clean injection supplies, respectively–are not key ingredients in the production of the harms of injection drug use [31, pp. 355–357].

For present purposes, I must first note that Kirschenheiter and Corvino’s arguments do not pertain directly to safer supply; even if they are right on all counts, safer supply might be unlike SISs and NEPs in enabling, facilitating, or condoning immoral drug use. But the argument at issue is implausible for SISs and NEPs, and doubly so when extended to safer supply. The problem is with the claims that these services are not enabling or facilitating. Kirschenheiter and Corvino argue that providing a safe site or clean supplies for injection drug use does not contribute to the production of any harms that would not already come about if those services were not provided, and they liken such services to Red providing Pat a ballcap (an accidental accessory) rather than a gun (a key ingredient) [31, p. 356]. Tabling the concern that even providing an accidental accessory might entail complicity, it is hard to see how a space or supplies to inject drugs are not key ingredients to ensuing drug use and its harmful ramifications. As shown above in the example of Red providing Pat a gun, the issue is not whether someone who injects drugs can find other means of doing so, but how its harms are actually brought about. When a person overdoses injecting drugs with resources from an NEP or SIS, the HR program has clearly provided a key ingredient to this outcome rather than an accessory, and program staff bear some moral responsibility for it. The question is whether this moral responsibility has a negative valence, i.e., whether program staff are complicit in wrongdoing. If drug use is immoral, either because it is harmful or for some other reason, then service providers are culpable for participating in immoral behavior. This argument applies even more clearly to safer supply than NEPs and SISs, since safer supply provides drugs of addiction–not a ballcap or even a gun, but, as it were, ammunition that can be used even without a gun. It is worth noting that, since paradigmatic HR services enable harms, they generally seem to facilitate them as well. Even if HR programs reduce the prevalence of drug use at the population level, to provide a key ingredient in the production of a harm is normally to make that particular harm more likely to occur.

## Moral shielding

Another widespread objection to HR services is that they shield PWUD from the deserved negative consequences of their immoral behavior. On this view, the immorality of drug use makes it good when PWUD suffer its negative consequences, and bad when PWUD are protected from those negative consequences. On this view, the negative consequences of immoral behavior function as punishment.

Lindsay Brooke Porter argues that PWUD do not deserve many of the negative consequences of drug use, and that HR measures which shield PWUD from these consequences are warranted [33]. Porter doubts that there is any principled basis for thinking that drug use is immoral [33, p. 366], but gives two arguments to show that, even if it is, PWUD do not deserve many of its negative consequences. Their first argument is that many of the harms of drug use are not intrinsic to it, but products of extrinsic factors like punitive drug policies and social determinants of health. While PWUD may deserve the ‘natural’ consequences of their drug use–intoxication and withdrawal, perhaps–they do not deserve consequences that have been artificially piled onto it, for instance by restricting needle purchases for illicit drug use, or by making PWUD resort to black market drug supplies [33, pp. 364–365, 367]. Secondly, many of the harms of drug use are disproportionate to its putative degree of immorality. If drug use is immoral, surely it is not so immoral as to merit lifelong infection with HIV, or death from overdose [33, pp. 366–367]. Having argued that PWUD do not deserve negative consequences of drug use that are artificial or disproportionately bad, Porter then argues that it is unjust to allow those consequences to accrue to PWUD, giving rise to positive moral reasons for taking HR measures [33, pp. 369–370].

I am not convinced by Porter’s arguments. First consider the argument that PWUD do not deserve the artificial consequences of drug use, like HIV infection due to artificially restricted needle supply. If one thinks society ought to disincentivize drug use by making it riskier than it naturally is–perhaps because it has the potential to harm others as well as oneself–then one might also think that people who take the artificial risks of drug use deserve the artificial consequences. In this line of thinking, a person who goes out of their way to flout social norms or laws against drug use should not be rescued from the hostile conditions that exist beyond the rule of those norms, whatever those hostile conditions may be. One only gets the conclusion that artificial consequences of drug use are undeserved, qua artificial, when one denies that society should disincentivize drug use by making it riskier than it naturally is. That is rather close to the conclusion Porter aims to establish and many will find it easy to deny in the absence of further reasons to accept it.

Porter’s second argument holds that, even if drug use is immoral, it is not so immoral as to warrant consequences as severe as lifelong illness or death. There are two problems with this line of reasoning. Firstly, it is unclear why one should deny that drug use is sufficiently immoral to warrant severe consequences, if one cannot say why one supposes it is immoral. Relatedly, one must recognize the difference between the desert of unintended outcomes and the desert of intentional punishment. Even supposing that PWUD do not deserve capital punishment or intentional HIV infection for their drug use, this does not mean that the accidental occurrence of these consequences is unduly severe for someone who risks them. Illegal drag racers should not be put to death when caught, but death in a car accident is not a morally disproportionate consequence of drag racing.

## Addictive drug use in safer supply is not immoral

Recent arguments against complicity and moral shielding in the provision of HR are unconvincing. This reinforces the intuition that it is immoral to make immoral behavior easier or less costly for others. The moral quality of safer supply and other HR services does, in fact, appear to depend on the moral quality of the behaviors they support. If the drug use supported by an HR service is immoral, then so, it would appear, is the service.

In what follows, I will evaluate the behavior that safer supply supports, to assess whether it passes moral culpability on to facilitators. Three methodological points to start: (1) While it can be tempting to say that all HR services just support the same “immoral drug use,” this is too quick. What safer supply facilitates is the legal, medically managed use of pharmaceuticals, over and against the illegal, minimally supervised, nonmedical use of street drugs that occurs outside of HR, and in HR services like SISs and NEPs. I will show that these two kinds of drug use differ in ethically significant ways. Secondly, since I do not mean to say that safer supply or other HR practices are suitable for all PWUD, I will focus on the ethical permissibility of drug use motivated by addiction, i.e. addictive drug use, which again, is ethically different from nonaddictive drug use. Thirdly, I will evaluate the actions under consideration by examining the two features commonly used for this purpose in ethical theory: motives and consequences. I will argue that the addictive drug use facilitated by safer supply does not have immoral motives, and that safer supply is recommended by consequentialist analysis. Indeed, safer supply is on firmer ground than some other HR interventions by the lights of general moral considerations.

Many believe that addictive behavior is immoral in virtue of what motivates it. They think that when people with drug addictions use drugs, they are selfishly seeking pleasure while carelessly harming others, which is immoral [34]. But that is not how addiction really works. While addiction is sometimes the result of pleasure-seeking drug use, once a person is addicted, they have become so severely physiologically or psychologically dependent that what they are addicted to is experienced as something without which they cannot live or function. For an addicted person who is not far along in recovery, to be deprived of what one is addicted to is extremely painful, even unbearably so [35]. While addictions and the people who suffer from them are highly heterogeneous, people with severe SUDs are never just happily flouting their responsibilities to abuse drugs. Rather, they are using drugs to maintain a minimal level of well-being and functionality, because complete cessation seems too painful, too disruptive, or too dangerous [36]. This is not an immoral motive [37, p. 20], and when viewed with charity, can in some cases be seen as prudent or virtuous.

When considering the consequences of addictive substance use, one must distinguish between the consequences of the illicit nonmedical substance use that safer supply is meant to displace, and the licit medical substance use that it facilitates. Safer supply is a harm-reducing intervention precisely because it eliminates or mitigates some of the worst consequences of addictive drug use for the PWUD and the people around them. Outside of a program like safer supply, nonmedical drug use typically involves the use of illicit drugs that must be procured through black market channels and cannot be paid for by an insurer. These factors give the drug user reasons to participate in the black market as buyers and to commit property crimes and acts of violence for the purpose of procurement, which harm others and society. Both illicit drug possession and use, and various forms of participation in the black market, put illicit drug users at legal risk, including risk of arrest, prosecution, conviction, and incarceration. These legal consequences harm the drug user directly and their community indirectly.

Similarly, the illicit drug use that occurs outside of safer supply puts drug users at great risk of adverse health outcomes like overdose and death, which harm loved ones almost as much as the drug user themself. In comparison to illicit nonmedical drug use, the drug use that safer supply facilitates is vastly superior by the consequentialist standard. Safer supply provides drugs that are legal for the user to possess and use. These drugs may be subsidized in part or whole by insurance, greatly reducing the incentive to commit harmful acts to fund procurement. Management of unadulterated pharmaceuticals by a medical provider greatly reduces the risk of negative health outcomes, saving lives and protecting the families and communities of PWUD. In short, the drug use facilitated by safer supply has relatively few negative consequences, many fewer than the behavior it replaces. Under properly administered safer supply, the consequences of addictive drug use primarily include intoxication, avoidance of withdrawal, and a level of general functionality that is lower than abstinence outside of addiction but greater than abstinence within it. HR is normally justified on consequentialist grounds [6], and this is why: HR services give service recipients and their communities access to better outcomes. Notably, the fact that HR services like SISs and NEPs facilitate illicit nonmedical drug use suggests that safer supply is on particularly firm moral ground among HR interventions.

It will be objected that the consequences of safer supply should not be compared to those of illicit nonmedical use, but to those of abstinence. Abstinence, the thought goes, reduces the harmful consequences of drug use more than the addition of medical supervision and the use of pharmaceuticals does. And some will think that it is impermissible to facilitate suboptimal outcomes; on several consequentialist views, one is obligated to act in a way that will maximize, rather than merely improve, good outcomes [38]. The main problem with this objection is that cessation of drug use is not straightforward in addiction. Abstinence in addiction is notoriously difficult. While addicts have some control over their addictive behavior, this control is reduced in degree and reliability, likely making long-term abstinence impossible for some addicts without the right kinds of assistance [39, 40]. Most addicts have earnestly tried to quit more than once and have repeatedly failed [41]. These points hold for a wide range of drug and non-drug addictions, all of which involve profound impairments in the psychological and neurobiological decision-making systems [42]. The ideal candidate for an HR service like safer supply is not someone who can clearly quit on their own, but someone for whom cessation and reduction programs have proven unsuccessful, inaccessible, or unlikely to succeed. Safer supply is a last-line intervention that can compare favorably with alternatives in extreme cases.

I have argued that the drug use supported by safer supply is not immoral in virtue of its motives or consequences. A couple of objections might still be raised to the argumentative strategy that I have pursued. Related to the idea that safer supply supports behavior with a bad motive is the deontological view that it enables, facilitates, or condones behavior that violates a duty against self-harm. Here, one might think that there is a moral duty not to harm oneself, and that the drug misuse supported by safer supply is immoral because it violates this duty, qua self-harming. This would make safer supply culpably complicit in immoral behavior. Deontologists do sometimes argue that intending certain forms of self-harm is immoral [43, 44], but principles about the morality of self-harm must be formulated very specifically to be plausible—and almost certainly in a way that permits safer supply. It is widely accepted as ethical to inflict harms on oneself to benefit oneself or others, both within and outside of healthcare. As I will discuss with regards to the principle of nonmaleficence below, most medical interventions are harmful, but justified in terms of greater goods for the patient. It is permissible and perhaps sometimes obligatory to receive chemotherapy, an intrinsically harmful kind of drug, to destroy cancer. Furthermore, it is often not just permissible, but supererogatory, to donate an organ to save someone else. Outside of medicine, it can be permissible, obligatory, or supererogatory to deprive oneself of sleep or food, or to expose oneself to injury, in service of a higher good such as academic or athletic excellence; consider, for instance, Marie Curie exposing herself to radioactive substances for science. And it is certainly at least permissible to jump on a grenade or otherwise sacrifice oneself for the benefit of others. Notably, safer supply benefits not just the patient but their loved ones, their community, and society, and will often be undertaken for its manifold holistic benefits, and not just for the benefits that accrue to the patient. In light of these considerations, the drug use supported by safer supply does not appear to be ruled out against principles forbidding self-harm. Keeping to the concern with motive that characterizes deontological ethics, this is because safer supply does not *aim* at harm but at benefits, and harms may simply be accepted as foreseen but unwanted adjuncts. This comports with the famous ‘doctrine of double effect,’ according to which it can be permissible to incur foreseen harms when the primary intent of action is good.

Another objection to the ethical assessment of addictive drug use I have provided is that it neglects perspectives from virtue ethics. Virtue ethics theories exhort one to cultivate good character traits through the practice of good deeds, as means to overall personal excellence, flourishing, and happiness [45]. Substance misuse reflects a deficit in the classical Aristotelian virtue of temperance [46], and addiction might be understood as the habituation of the corresponding vicious excess of self-indulgence. One might therefore argue that safer supply enables, facilitates, or endorses vice rather than virtue, entailing culpable complicity. The absence of virtue theoretic analyses of HR in general, and safer supply in particular, make it difficult to engage deeply with this line of thought. But safer supply has been shown to help the worst afflicted patients to stabilize their lives and focus on basic needs, enabling them to escape from street life, establish permanent housing and work, and achieve overall higher levels of societal integration and personal flourishing [4]. On the face of it, then, safer supply appears to promote the cultivation of virtue more than vice, even if it does not cultivate the virtue of temperance directly.

The drug use enabled in well administered safer supply is not immoral. Providers of safer supply are partly responsible for the drug use enabled by their programs, but they are not complicit in wrongdoing in a way that is morally problematic. Furthermore, these programs do not shield participants from negative consequences that might be deserved in virtue of their immoral conduct. Rather, they shield participants from the negative consequences of nonmedical drug use by largely replacing it with medical use, which is ethically permissible. The ethical permissibility of addictive drug use within safer supply diffuses the most pressing general moral objections to the practice.

## Objections from biomedical ethics

As a medical intervention, the most challenging objections to safer supply will come not from general moral considerations, but from ethical norms internal to healthcare professions. I will address the following objections from biomedical ethics in turn: (a) Safer supply harms patients, contrary to the principle that medical providers ought to “do no harm;” (b) safer supply facilitates the unhealthy choices and lifestyles of patients; and (c) addicts cannot provide informed consent for safer supply.

### Nonmaleficence

According to the famous Hippocratic injunction “Primum non nocere,” physicians ought not harm patients. This is commonly called ‘the principle of nonmaleficence’ and gets extended to all healthcare professionals and medical researchers [25, ch. 5]. One might object to safer supply that it harms patients, perhaps by contributing to the harms that result from intoxication, deepening their addictions, or exposing them to risk of overdose.

In fact, most medical interventions do harm, even when successful. Drawing blood causes mild discomfort; antidepressant medications cause weight gain; open-heart surgery causes severe injury; chemotherapy causes nausea, fatigue, and so on. But these interventions can be justified by the probabilities of the positive health outcomes at which they aim. The principle of nonmaleficence demands that harms brought about in healthcare be justified by prospective overriding health benefits, and moreover, that harming the patient is never the ultimate goal. It does not require that healthcare professionals literally do no harm. As Jonsen, Siegler, and Winslade say,

It would clearly be wrong for a physician to set out to harm a patient, but it is almost inevitable that when a physician attempts to benefit a patient, for example by medication or surgery, that some harm or risk of harm is possible or may ensue… A physician must calculate [the risk-benefit ratio of an intervention] and fashion it into a recommendation to the patient [47, p. 13]

Major topics of controversy around which nonmaleficence is invoked include the withholding and withdrawal of life-sustaining treatment, physician-assisted suicide, and the use of health information for potentially harmful research. Safer supply and other HR interventions are not paradigms of potentially maleficent medicine.

While safer supply puts patients at some risk, its primary goal is to prevent serious negative health outcomes. A medical provider might reasonably judge that reducing the risks of HIV infection, overdose, and addiction to adulterants outweigh the risks of intoxication and worsened dependence, which are likely to occur in nonmedical use anyhow, and which the provider has no power to prevent. As a form of HR, safer supply is justified to the extent that it promotes better health outcomes for the patient overall. Indeed, closely related to the principle of nonmaleficence is the principle of beneficence, mentioned above, which commits healthcare professionals to promoting patients’ best interests by maximizing benefits and minimizing harms. Safer supply aims to prevent bad health outcomes for the patient, to remove harms associated with their risky behavior, and to give them a happier and healthier life. The principle of beneficence justifies safer supply.

## Facilitating an unhealthy lifestyle

Another concern about HR practices like safer supply is that they facilitate the unhealthy lifestyles of patients. This is a variation on the moral objection that HR practices facilitate the immoral behavior of people with drug addictions. One might think that, just as healthcare professionals have obligations to maximize benefits and minimize health-related harms to patients, they have obligations not to make it easier for patients to engage in unhealthy activities themselves. But, the thought goes, HR interventions do make it easier for people with drug addictions to engage in unhealthy drug use.

While this may appear a natural way to specify the principles of beneficence and nonmaleficence, in fact, healthcare professionals have no obligation to withhold care that would make an unhealthy lifestyle easier or less costly for the patient. Leading causes of disease, illness, and death in the developed world, including heart disease, cancer, and diabetes, are often due to lifestyle. Any interventions that enhance health or longevity for patients with these conditions and which are not premised on the cessation of unhealthy behaviors, facilitate an unhealthy lifestyle. Indeed, healthcare professionals are not just permitted to provide such interventions in typical cases, but are often obliged to. Consider the case of the patient with diet-related hypercholesterolemia who struggles to modify her diet. If statins are medically indicated to lower her cholesterol and reduce her risk of heart attack and stroke, then it would be unethical for her physician to withhold that medication based on her lifestyle. Then there is the case of the sex worker who has contracted a sexually transmitted bacterial infection on the job. It would be wrong for her provider to withhold antibiotics, even though they know that this will hasten her return to work, which comes with serious health risks. As a third example, consider the case of the diabetic patient who requires an amputation due to life-threatening gangrene. It would be unethical to withhold this surgery even if the patient plainly refuses to comply with lifestyle changes that will mitigate the harms of diabetes.

What these examples illustrate is that HR, broadly construed, is integral to healthcare and the values that govern it. In everyday life, enabling or facilitating immoral or imprudent behaviors of others is generally considered wrong. But as an endeavor that strives to be “value-free” in certain respects [48], healthcare is committed to promoting patient health and public health, even when this can be seen as enabling or facilitating unhealthy behavior. It is not a healthcare professional’s place to say that an intervention is not deserved or indicated because the patient engages in medically risky behavior; such judgments call into question the value of indisputably preferable health outcomes, a foundational premise of healthcare. Concerns about facilitating unhealthy lifestyles are therefore moot, as objections to safer supply or any other healthcare intervention.

## Informed consent

A tenet of biomedical ethics is that medical providers must not provide an indicated intervention without first securing informed consent from the patient, whenever possible. To provide informed consent, a patient must have ‘decision-making capacity’ (‘capacity’). To have capacity is to have a mental status in which one can be responsible for one’s medical choices. Conditions like intoxication, unconsciousness, and intellectual disability can compromise capacity. Whether addicts have capacity to provide informed consent for safer supply has been the subject of some scholarly debate. If not, then this intervention would appear to be ethically impermissible, at least without the consent of a surrogate.

Debate around capacity and heroin prescription has focused on whether addiction is compulsive. On one side, authors like Louis Charland have argued that because addiction is compulsive, people who are addicted to heroin are not competent to consent to its prescription [49]. For Charland, addictive compulsion means that one’s stable values have been displaced by an inflated care for drugs [42–44], which makes it impossible for the addicted person to rationally assess the risks and benefits of heroin maintenance trials or therapy. The ability to rationally manipulate information about a treatment is a requirement for the capacity to consent according to the widely accepted MacArthur model of competence [50], leading Charland to conclude that people addicted to heroin do not have capacity [49, p. 42]. On the other side, authors like Bennett Foddy and Julian Savulescu have argued that addiction is not a compulsion, and that people addicted to heroin do have capacity to consent to heroin prescription [51]. For Foddy and Savulescu, addictive desires or cravings are ordinary appetites [51, pp. 9–12]—presumptively authentic ones [51, pp. 12–14]—which addicted persons are able to resist when they have reason to [51, pp. 5–6]. Furthermore, the opportunity to participate in safer supply or a heroin prescription trial does not deprive people addicted to heroin of maintaining their status quo of nonmedical use and is therefore not coercive [51, pp. 6–8]. Having found no basis to think addiction is compulsive, Foddy and Savulescu conclude that addicted persons have capacity [51, p. 14].

Both of the above arguments get addiction wrong in significant ways. Charland is wrong that people with addictions do not have stable values. While addictive behavior is often self-destructive, addicted persons do not stop valuing universally esteemed goods, including life, longevity, health, bodily integrity, freedom from pain, companionship, and financial independence. The xylazine user may inject this drug despite the horrible wounds it causes, but he desperately wishes that it did not cause these wounds and will seek out care because he does not want to lose life or limb. Furthermore, the fact that this person has and acts on conflicting values (as most do) does not entail that he is incapable of rationally comparing related options. Foddy and Savulescu dramatically oversimplify addiction by ignoring its autonomy-related features other than craving [52], and by ignoring the ways in which addictive cravings differ from ordinary ones, even just in terms of intensity, duration, and recurrence [53]. As I explained above, addiction is a distinctive form of physiological and psychological dependence. While people with addictions clearly have control over much of their addictive behavior, this control is distinctively unreliable [54, 55], in a way that is profoundly disruptive and putatively compulsive [39, 40].

Addiction’s putative compulsivity does not make informed consent to safer supply impossible, but it does necessitate consent-protecting measures. Addiction compromises a person’s ability to abstain from what they are addicted to, or put otherwise, one’s ability to choose between using and not using. It does not, however, compromise one’s ability to choose among comparable sources or forms of what one is addicted to. If a person with a severe, active heroin addiction were presented with the choice between a smaller bag of more potent heroin and a larger bag of less potent heroin (identical in morphine equivalency, say), their addiction would not dictate one choice or the other. Likewise, if this person were presented with the choice between medically managed access to pharmaceutical heroin that would replace some nonmedical street heroin use, and a greater share of nonmedical street heroin use, their addiction would not compel them to consent to the former. When one recognizes that, contra Charland, people want to be protected from the harms of an unsafe drug supply whether they have an addiction or not, it seems obvious that consent to safer supply normally reflects those values rather than compulsion. There is one way in which otherwise comparable alternatives can differ so as to affect one’s ability to choose between them in addiction: how drug use is timed. People with addictions do discount the value of future rewards more than usual when those rewards are being compared against more immediate drug use, a phenomenon referred to as ‘hyperbolic discounting’ [56]. So, an addicted person might be compelled to choose a lower morphine equivalency of heroin that they can use now, over a vastly greater morphine equivalency they have to wait a day to use. This feature of addiction ought to be considered when determining how to administer safer supply, so as to minimize its role in patient decision-making.

Aside from the issue of hyperbolic discounting, Foddy and Savulescu note two specific practices that protect informed consent in prescribing drugs of addiction. Consent should be secured when the candidate is (a) not severely intoxicated, and (b) not in severe withdrawal. Intoxication and withdrawal cannot compel consent to treatment set sufficiently far in the future; but they can impair a person’s ability to rationally manipulate information about the treatment on offer. So, while safer supply is often in the best interest of the patient, providers should bear in mind that aspects of addiction can undermine decision-making capacity and therefore the ability to provide truly informed consent.

## Conclusion

Safer supply is an evidence-supported, last-line medical intervention for treatment-refractory drug addiction. I have argued that, in addition to being safe and effective when medically indicated, safer supply can also be ethically permissible. Whatever the ethical status of the nonmedical drug use it displaces, the drug use supported by safer supply is not immoral. Furthermore, safer supply conforms to the established ethical standards of the healthcare professions. In the final analysis, its differences from more conventional forms of harm reduction–and indeed, more conventional medical care–are largely superficial.

Despite being fairly conventional medicine, safer supply is not widely advocated in healthcare or by harm reduction organizations. Given what a serious public health problem addiction is in so many parts of the world, it is worrisome that a sound intervention is not utilized and promoted more. Ethical considerations should inform how safer supply is provided and stoke further scientific research, rather than quash interest in it. Insofar as safer supply is seen as too politically controversial to practice or promote, I hope that harm reduction advocates will arm themselves with my arguments, which highlight best practices that keep safer supply in line with other forms of harm reduction and more familiar medical interventions. Safer supply can save and improve lives when it is properly implemented.
